# Different types of mobile phone use while driving and influencing factors on intention and behavior: Insights from an expanded theory of planned behavior

**DOI:** 10.1371/journal.pone.0300158

**Published:** 2024-03-06

**Authors:** Hassan Okati-Aliabad, Raheleh Hashemi Habybabady, Mohammad Sabouri, Mahdi Mohammadi

**Affiliations:** 1 Health Promotion Research Center, Zahedan University of Medical Sciences, Zahedan, Iran; 2 Student Research Committee, Zahedan University of Medical Sciences, Zahedan, Iran; Al Mansour University College-Baghdad-Iraq, IRAQ

## Abstract

Mobile phone use while driving (MPUWD) is a significant concern due to its negative impact on road safety. This cross-sectional study aimed to assess different types of MPUWD and identify factors influencing intention and behavior among drivers in Zahedan. A total of 392 participants provided information on demographic and driving characteristics, as well as constructs derived from the Theory of Planned Behavior (TPB) augmented with moral norms. Findings revealed that a majority of drivers (86.4%) engaged in MPUWD, primarily involving calling, using applications, and texting. However, most participants did not intend to use their phones while driving in the following week. Multiple regression analysis identified age, receiving driving fines, subjective norm, and perceived behavior control as significant predictors of intention for MPUWD. Additionally, factors such as age, receiving driving fines, driving hours, car gearbox type, attitude, perceived behavior control, behavioral intention, and moral norm were significant predictors of actual MPUWD. Older participants demonstrated better behavior in various mobile phone activities while driving. Overall, the study emphasized that the main TPB constructs and moral norms have a greater influence compared to other variables in predicting MPUWD. Perceived behavioral control was the most important predictor of the intention and behavior of MPUWD. Understanding these factors can guide efforts to discourage MPUWD through targeted interventions and strategies to promote safer driving practices.

## 1. Introduction

### 1.1. Distracted driving

The increasing prevalence of distracted driving has raised significant concerns regarding road safety on a global scale [1]. Distracted driving encompasses various activities that draw a driver’s attention away from the essential task of safely operating a vehicle [2]. Distracted driving involves a range of distractions, including visual, cognitive, and manual elements. Visual distractions occur when drivers shift their gaze away from the road to focus on objects, events, or tasks inside or outside the vehicle. Cognitive distractions arise when drivers’ thoughts or activities unrelated to driving occupy their minds, leading to compromised focus, attention, and decision-making abilities. Manual distractions occur when drivers engage in physical tasks or actions that require contact or manipulation [3]. Research has consistently shown a significant increase in the likelihood of accidents when drivers are distracted while driving [4, 5]. Moreover, distracted driving substantially raises the risk of committing traffic violations [6], resulting in more severe accidents compared to undistracted driving [7].

### 1.2. Mobile phone use while driving (MPUWD)

MPUWD has emerged as a significant concern due to the widespread use of smartphones among mobile phone users [8]. Previous on-road observations have identified mobile phone use as the primary form of distraction while driving [9]. It is well-established that driver distraction, especially from cell phone use, contributes significantly to crashes [4, 10]. Research indicates that the use of smartphones while driving disrupts normal driving behavior, resulting in sudden steering changes, reduced concentration, and increased cognitive load [11]. Numerous studies have investigated the impact of MPUWD on driving performance, including reaction time, speed, lane positioning, and collision risk [12–14]. Engaging in MPUWD impairs drivers’ ability to effectively divide their attention and maintain concentration, posing a substantial risk of life-threatening traffic incidents [15]. Drivers who frequently engage in MPUWD have been observed to demonstrate accelerated driving speeds, frequent lane changes, prolonged occupancy of the left lane, as well as an increased occurrence of hard braking and rapid acceleration. Furthermore, they report more traffic violations and display more favorable attitudes toward speed and passing compared to drivers who do not regularly use cell phones while driving [16].

## 2. Literature

### 2.1. Factors influencing intention and behavior of MPUWD

Numerous studies have examined the factors that influence drivers’ intentions and behaviors regarding MPUWD. Research indicates that individual factors, including age [17], receiving driving fines [18], and driving hours [19], play a significant role in the likelihood of engaging in phone use while driving. Understanding these determinants is crucial for the development of effective interventions and policies aimed at promoting safer driving practices and reducing the prevalence of MPUWD.

### 2.2. The theory of planned behavior

To gain insights into the factors influencing intention [20, 21] and behavior [22, 23] of MPUWD, researchers have explored psychological frameworks such as the theory of planned behavior (TPB). The TPB proposes that attitude, subjective norms, and perceived behavioral control collectively shape an individual’s intentions and subsequently influence their actual behavior [24]. Attitude refers to an individual’s evaluation, which can be either positive or negative, of a behavior. It encompasses their beliefs about the anticipated outcomes or consequences of that behavior and their overall assessment of its desirability [25]. Evidence indicates that a positive attitude toward using a mobile phone while driving enhances both the intention and behavior of phone use [23, 26]. Subjective norm, as a construct in the TPB, refers to individuals’ perceptions of social pressure or influence from significant others regarding a specific behavior. It reflects individuals’ beliefs about whether others think they should or should not engage in a particular behavior based on social norms and expectations [27]. Previous studies suggest that subjective norms were not significant predictors of intentions to send or read text messages and monitor or read social interactive technology while driving [20, 28]. Perceived behavioral control entails an individual’s perception of their ability to perform a behavior and their confidence in overcoming obstacles or constraints. It encompasses factors such as perceived confidence, available resources, opportunities, and skills needed to engage in the behavior successfully [29]. A survey on mobile phone use intentions while driving among public service vehicle drivers found that perceived behavioral control was significantly negatively correlated with intentions to use a mobile phone while driving. Additionally, perceived behavioral control emerged as the strongest predictor of drivers’ intention to use a mobile phone [30]. Behavioral intention, a crucial component of the TPB, refers to an individual’s readiness and willingness to engage in a specific behavior. It represents their conscious decision and level of commitment to perform the behavior in the future [31]. In young drivers, intention was identified as the sole significant predictor of behavior when responding to social interactive technology on a smartphone in a concealed manner [32]. While the TPB offers a comprehensive framework, additional factors are essential for a comprehensive understanding of MPUWD. One such factor is the moral norm, which delves into the ethical dimensions of MPUWD [33, 34]. The moral norm encompasses an individual’s perception of the moral rightness or wrongness of behavior [24].

### 2.3. The current study

Given the growing concern surrounding MPUWD, this study aims to investigate different types of MPUWD and examine the factors influencing the intention and behavior of general drivers in Zahedan, Iran. Drawing upon the Theory of Planned Behavior (TPB) framework and emphasizing the role of the moral norm, this research provides valuable insights into the cultural and contextual factors that influence MPUWD. Unlike many previous studies that primarily focus on developed countries, this study takes a comprehensive approach by simultaneously examining both intention and behavior related to MPUWD. It goes beyond traditional behaviors such as calling and texting to explore a wide range of mobile phone usage behaviors while driving, providing a more nuanced understanding of the phenomenon. In addition to examining mobile phone usage behaviors, the study considers various factors, including socio-demographic characteristics and driving characteristics. It also incorporates key theoretical constructs from the TPB, such as attitude, subjective norms, perceived behavioral control, and intention, to comprehensively explore the determinants of MPUWD. Importantly, the study also recognizes the significance of the moral norm as a factor influencing both intention and behavior. Based on this foundation, the study formulated several hypotheses to guide the investigation.

Hypotheses:

H1. Attitude, subjective norms, perceived behavioral control, and moral norms predict MPUWD intention and behavior.
H2. With increasing age, participants report lower MPUWD intention and behavior.
H3. After adjusting for significant socio-demographic and driving characteristics, attitude, subjective norms, perceived behavioral control, and moral norms remain predictors of MPUWD intention and behavior.

## 3. Material and methods

### 3.1. Participants and procedure

A total of 392 drivers took part in this cross-sectional study. To determine the sample size, a pilot study was conducted with 30 drivers. Considering a significance level (α) of 0.05, a proportion of frequent use of mobile phones while driving at 35%, and an estimated error of 0.05, the sample size was estimated to be 350 individuals. Ultimately, 392 individuals participated in the study. The inclusion criteria for participants in this study required them to meet several criteria, including owning or using a cell phone, having a minimum of one year of driving experience, being above 18 years of age, currently driving, and providing informed consent. Data collection took place in January-February 2023 at Zahedan fuel stations in southeast Iran during morning and afternoon periods. Given Zahedan’s role as the capital of Sistan and Baluchistan province, drivers frequent various fuel stations along routes to the north and south. Zahedan exhibits diverse cultural and social contexts across its different parts. Hence, for comprehensive sampling of a diverse and representative population, data collection was conducted at all 10 gas stations in Zahedan. For the data collection process, we employed a team of four trained individuals. Their training included guidance on approaching participants, explaining the study’s objectives, and ensuring the confidentiality of the collected information. Additionally, they were briefed on potential challenges they might encounter and the appropriate procedures for addressing them.

### 3.2. Measures

Data were collected through a self-administered questionnaire that covered various aspects. The questionnaire included socio-demographic characteristics, driving characteristics, TPB constructs (attitude, subjective norms, perceived behavior control, and intention), moral norms, and behavior. The validity and reliability of the questionnaire were confirmed utilizing content validity and internal consistency reliability. To establish content validity, a panel of experts in health behavior and traffic accidents meticulously investigated the questionnaire. The expert panel assessed each item for relevance, representativeness, and clarity, with subsequent adjustments made based on their recommendations. The questionnaire’s reliability was ensured through a pilot test involving 30 individuals similar to the target respondents. All subscales demonstrated acceptable Cronbach’s alpha coefficients (≥0.70).

Socio-demographic characteristics encompassed age, gender, marital status, and education. Driving characteristics included driving license, certificate type, gearbox type, driving experience, accidents and driving fines in the last year, driving hours, and driving purpose.

In Iran, driving licenses are categorized into three grades, each with its age requirements. The minimum age for obtaining a first-grade license is 18 years, while for a second-grade license, it is 23 years, and for a third-grade license, it is 25 years. Additionally, there are specific restrictions associated with each license grade. Third-grade licenses are issued for driving motor vehicles with a seating capacity of up to 9 passengers and vehicles weighing up to 3500 kg gross vehicle weight (GVW). Second-grade licenses allow for driving motor vehicles with a seating capacity of up to 26 passengers and vehicles weighing up to 6000 kg GVW. Finally, a first-grade license is required for driving buses and trucks with a capacity exceeding 6000 kg GVW.

The TPB questionnaire was developed by the research team according to Ajzen [35, 36] and exhibited strong internal consistency with Cronbach’s alpha values of at least .80 for all TPB constructs. Attitude towards MPUWD was measured using a single statement “For me using a mobile phone while driving would be” followed by five items semantic differential scales. Items were scored from (1) safe to (7) dangerous; (1) beneficial to (7) harmful; (1) good to (7) bad; pleasant (1) to unpleasant; non-stressful (1) to stressful (7). The total attitude score ranged from 5 to 35 and a higher score means a more negative attitude toward MPUWD.

Subjective norm was measured with three items, “Most of the important people in my life want me to use my cell phone while driving”, “Most of the important people in my life approve me to use a cell phone while driving”, “Most of the important people in my life think I should use my cell phone while driving”, Questions were answered on a 5-point Likert scale from strongly agree 1 to strongly disagree 5. The total subjective norm score ranged from 3 to 15 and a higher score indicates disapproval of MPUWD by significant others.

Perceived behavior control was measured with 3 items, “I have complete control over my cell phone usage while driving”, “I am confident I can use my cell phone while driving”, “It is easy for me to use my mobile phone while driving”, Questions were answered on a 5-point Likert scale from strongly agree 1 to strongly disagree 5. The total perceived behavior control score ranged from 3 to 15 and a higher score indicates lower perceived behavior control.

Moral norm was measured using three items adapted from Nemme and White [37], “I feel guilty if I use my cell phone while driving”, “I personally think that using a cell phone while driving is wrong”, “Using a cell phone while driving is against my principles”, Questions were answered on a 5-point Likert scale from strongly disagree 1 to strongly agree 5. Moral norm items were translated into Persian, and a cross-cultural evaluation and test adaptation approach was employed, guided by the committee’s considerations [38]. The total moral norm score ranged from 3 to 15 and a higher score indicates a stronger moral norm.

The intention to engage in MPUWD was measured with three items, “I plan to use my cell phone while driving next week”, “I will most likely be using my cell phone while driving in the next week”, “I expect to use my cell phone while driving in the next week”, Questions were answered on a 5-point Likert scale from strongly agree 1 to strongly disagree 5. The total intention score ranged from 3 to 15 and a higher score indicates lower levels of intention.

The behavior section consisted of 16 questions that investigated engagement in MPUWD, answering phone calls, making phone calls, reading messages, sending messages, reading emails, sending emails, reading/viewing social media posts, sending social media posts, answering video calls, making video calls, using phone applications, taking a picture, turning off the phone, stop driving to answer received phone calls or messages, and using headphone. Questions were answered on the following scale: 1 = always, 2 = most of the time, 3 = sometimes, and 4 = never. The total scores of behavior ranged from 16 to 64 and a higher score indicates lower levels of MPUWD.

The study protocol obtained approval from the Ethical Committee of Zahedan University of Medical Sciences (IR.ZAUMS.REC.1401.081). Before administering the questionnaire, the researcher provided a comprehensive explanation of the study’s objectives and obtained written informed consent from the participants, ensuring their voluntary participation.

### 3.3. Statistical analysis

Quantitative variables were reported as mean ± standard deviation (SD), while qualitative variables were presented as frequencies (%). The associations between demographic factors and driving characteristics with behavior and behavioral intention were analyzed using one-factor and multi-factor univariate general linear models. The relationships between the TPB constructs and moral norms with behavior and behavioral intention were assessed using Pearson correlation and multiple regression models. One-way analysis of variance was used for mean comparison of behavior in terms of age and test for trend. Statistical analyses were carried out using IBM SPSS Statistics 24, and a significance level of 0.05 was used to determine the statistical significance of the results.

## 4. Results

### 4.1. Socio-demographic characteristics

A total of 392 drivers with a mean age of 36.30 ± 11.01 years took part in this study. The majority of participants were male (66.2%), married (69.1%), and university graduates (66.2%). Approximately two-thirds of participants had up to 15 years of driving experience, around 15% worked by car, and 61.8% drove for more than one hour per day. Only 38.6% had not received any driving fines, and 31.3% had experienced at least one accident in the past year. The majority of participants (91.3%) owned a smartphone, with the primary use being for making calls while driving (93.8%). A significant proportion of participants (63.8%) expressed a preference for using a mobile phone while driving instead of hands-free or other devices. Additionally, 26.4% of participants reported being involved in accidents due to MPUWD and 39.1% had received penalties for engaging in MPUWD.

### 4.2. Frequency of MPUWD

A significant majority of participants (86.4%) reported engaging in MPUWD. The most common activities included answering phone calls (86.4%), making phone calls (76.9%), using phone applications (68.4%), and reading messages (53.2%). Additionally, a considerable number of participants reported sending messages (41.4%). On the other hand, fewer participants were involved in reading emails (10.3%), sending emails (9.3%), reading/viewing social media posts (29.6%), sending social media posts (23.2%), answering video calls (26.3%), making video calls (21.5%), and taking pictures (35.4%) while driving. Moreover, 26.1% of participants reported turning off their phones, 42% used headphones, and 34.8% frequently stopped their driving to answer phone calls or messages (Table 1).

**Table 1:** Frequency distribution of behavior items.

	Always	Usually	Sometimes	Never
Using a mobile phone while driving	28 (7.2)	94 (24.1)	215 (55.1)	53 (13.6)
Answering phone calls while driving	81 (20.8)	92 (23.6)	164 (42.1)	53 (13.6)
Making phone calls while driving	47 (12.2)	71 (18.4)	179 (46.4)	89 (23.1)
Reading messages while driving	20 (5.2)	47 (12.1)	139 (35.9)	181 (46.8)
Sending messages while driving	16 (4.2)	34 (8.9)	109 (28.4)	225 (58.6)
Reading emails while driving	9 (2.3)	5 (1.3)	26 (6.7)	348 (89.7)
Sending emails while driving	7 (1.9)	6 (1.6)	22 (5.8)	342 (90.7)
Reading /viewing social media (e.g., Instagram, WhatsUp, Telegram) posts while driving	11 (2.8)	20 (5.1)	84 (21.6)	274 (70.4)
Posting on social media (e.g., Instagram, WhatsUp, Telegram) while driving	11 (2.8)	19 (4.9)	60 (15.5)	298 (76.8)
Answering video calls while driving	17 (4.4)	23 (5.9)	62 (16.0)	286 (73.7)
Making video calls while driving	12 (3.2)	28 (7.4)	41 (10.9)	296(78.5)
Using phone applications (e.g., map) while driving	26 (6.7)	78 (20.2)	160 (41.5)	122 (31.6)
Taking pictures of yourself, others, or scenery while driving	11 (2.8)	25 (6.5)	101 (26.1)	250 (64.6)
Turning off the phone while driving	7 (1.8)	12 (3.1)	82 (21.2)	286 (73.9)
Stop driving to answer received phone calls or messages	46 (11.9)	88 (22.9)	159 (41.3)	92 (23.9)
Using headphones while driving	13 (3.4)	41 (10.7)	107 (27.9)	222 (58.0)

### 4.3. Age-related trend in MPUWD

There was an observed age-related trend in behaviors. Older individuals were found to be less likely to engage in certain mobile phone activities while driving, including using a mobile phone (p = 0.003), answering phone calls (p < 0.001), making phone calls (p < 0.001), reading messages (p = 0.002), sending messages (p = 0.004), reading/viewing social media (p = 0.014), posting on social media (p = 0.042), making video calls (p = 0.021), using phone applications (p < 0.001), and taking pictures (p = 0.001). On the other hand, older individuals were more likely to turn off their phones while driving (p = 0.012) (Table 2).

**Table 2:** Behavior of participants in mobile phone activities while driving based on age groups.

Age (year)	≤25	26–35	36–45	>45	P_value	P_value for trend
	Mean ± SD	Mean ± SD	Mean ± SD	Mean ± SD		
Using a mobile phone while driving	2.72±0.71	2.63±0.78	2.69±0.79	3.11±0.68	<0.001	0.003
Answering phone calls while driving	2.33±1.01	2.37±0.98	2.42±0.88	2.93±0.92	<0.001	<0.001
Making phone calls while driving	2.58±0.97	2.72±0.90	2.70±0.93	3.31±0.78	<0.001	<0.001
Reading messages while driving	3.10±0.81	3.16±0.79	3.26±0.94	3.53±0.82	0.011	0.002
Sending messages while driving	3.33±0.80	3.33±0.74	3.38±0.94	3.74±0.67	0.004	0.004
Reading emails while driving	3.82±0.54	3.87±0.52	3.77±0.66	3.89±0.46	0.441	0.687
Sending emails while driving	3.88±0.46	3.86±0.53	3.80±0.60	3.90±0.42	0.601	0.993
Reading /viewing social media (e.g., Instagram, WhatsUp, Telegram) posts while driving	3.57±0.76	3.46±0.72	3.64±0.72	3.82±0.56	0.005	0.014
Posting on social media (e.g., Instagram, WhatsUp, Telegram) while driving	3.62±0.82	3.59±0.69	3.65±0.69	3.85±0.57	0.077	0.042
Answering video calls while driving	3.59±0.74	3.50±0.85	3.57±0.82	3.81±0.57	0.061	0.084
Making video calls while driving	3.61±0.77	3.52±0.80	3.68±0.74	3.88±0.56	0.013	0.021
Using phone applications (e.g., map) while driving	2.78±0.86	2.84±0.87	2.96±0.89	3.44±0.76	<0.001	<0.001
Taking pictures of yourself, others, or scenery while driving	3.37±0.71	3.43±0.80	3.59±0.69	3.74±0.65	0.007	0.001
Turning off the phone while driving	3.87±0.34	3.65±0.66	3.65±0.64	3.59±0.70	0.057	0.012
Stop driving to answer received phone calls or messages	2.85±0.90	2.83±0.88	2.76±0.98	2.64±1.04	0.495	0.167
Using headphones while driving	3.28±0.90	3.29±0.87	3.35±0.79	3.78±0.48	<0.001	<0.001

### 4.4. Frequency distribution of behavioral intention items

The majority of participants (61.9%) did not intend to use their mobile phones while driving the next week, and a similar proportion (53.9%) did not expect to do so. Additionally, most individuals (53.5%) reported that they were unlikely to use their mobile phones while driving next week (Table 3).

**Table 3:** Frequency of behavioral intention items.

Behavioral intention items	Strongly agree	agree	Neither agree nor disagree	Disagree	Strongly disagree
	N (%)	N (%)	N (%)	N (%)	N (%)
I intend to use my mobile phone while driving next week	15 (3.9)	40 (10.4)	92 (23.8)	122 (31.6)	117 (30.3)
It is more likely to use my mobile phone while driving next week	23 (6.0)	62 (16.2)	93 (24.3)	108 (28.2)	97 (25.3)
I expect to use my mobile phone while driving next week	20 (5.2)	66 (17.2)	91 (23.7)	108 (28.1)	99 (25.8)

### 4.5. Age-related trend in behavioral intention

There was no observed age-related trend in behavioral intention (Table 4).

**Table 4:** Behavioral intention of participants regarding MPUWD based on age groups.

Age (year)	≤25	26–35	36–45	>45	P_value	P_value for trend
	Mean ± SD	Mean ± SD	Mean ± SD	Mean ± SD		
I intend to use my mobile phone while driving next week	3.25±0.98	3.41±1.06	3.49±1.18	3.56±1.17	0.412	0.095
It is more likely to use my mobile phone while driving next week	3.90±1.13	3.62±1.13	3.61±1.18	3.85±1.22	0.250	0.804
I expect to use my mobile phone while driving next week	3.52±1.11	3.46±1.08	3.72±1.18	3.74±1.16	0.211	0.134

### 4.6. Factors associated with MPUWD

The mean behavior score was 48.48 ± 7.44 and 37.8% of participants reported a lower level of MPUWD. In the one-factors model, the elderly (P<0.001), females (P = 0.040), married drivers (P = 0.022), and more experienced drivers (P = 0.006) used mobile phones less while driving. Additionally, fewer driving hours per day (P = 0.005), fewer driving fines (P = 0.001), and no experiencing accidents (P = 0.001) were significantly related to less MPUWD. Participants with a manual gearbox car also exhibited less MPUWD (P = 0.012) (Table 1). The mean score for behavioral intention was 10.78 ± 3.26, with 30.7% of individuals having a high negative behavioral intention. In the one-factor model, negative behavioral intention increased significantly with age (P<0.001) and driving experience (P<0.001). Conversely, negative behavioral intention decreased with accidents (P = 0.018) and driving fines (P = 0.004). Married drivers demonstrated a more negative behavioral intention compared to singles (P = 0.001) (Table 5).

**Table 5:** Relationship between demographic factors and driving characteristics with behavior and behavioral intention in the one-factor model*.

			Behavior		Behavioral intention		
		N (%)	Mean ± SD	P_value		P_value	
Age (year)				<0.001		<0.001	
	≤25	61(16.0)	47.20±7.00		9.72±3.36		
	26–35	134(35.1)	47.43±7.02		10.31±3.03		
	36–45	114(29.8)	48.40±7.82		11.04±3.30		
	>45	73(19.1)	51.90±6.66		12.15±3.01		
Gender				0.040		0.622	
	Male	259 (66.2)	47.91±7.77		10.89±3.26		
	Female	132(33.8)	49.56±6.66		10.71±3.26		
Education				0.068		0.174	
	under diploma	33(8.4)	51.28±6.73		11.90±3.04		
	Diploma	99(25.3)	48.79±6.14		10.86±2.99		
	BSc	173(44.2)	48.41±7.43		10.54±3.45		
	MSc & PhD	86(22.0)	47.23±8.81		10.76±3.18		
Marital status				<0.001		0.001	
	Single	114(29.1)	47.14±7.38		9.89±3.13		
	Married	271(69.1)	49.30±7.20		11.18±3.25		
	Divorced	7 (1.8)	39.14±9.28		9.71±2.93		
Driving license				0.180		0.335	
	Yes	387(99.0)	48.53±7.38		10.77±3.26		
	No	4(1.0)	43.50±13.30		13.00±2.83		
Driving certificate type				0.499		0.349	
	First grade	39(10.6)	48.21±7.53		11.44±2.71		
	Second grade	298(81.2)	48.53±7.47		10.64±3.29		
	Third grade	30(8.2)	50.10±5.49		10.77±3.33		
Gearbox type				0.012		0.097	
	Automatic	41(10.6)	45.78±10.70		9.95±3.69		
	Manual	345(89.4)	48.83±6.83		10.85±3.21		
Driving experience (year)				0.006		<0.001	
	≤5	86(22.3)	48.51±6.04		10.58±3.52		
	6–15	163(42.3)	47.20±7.68		10.04±3.20		
	16–25	94(24.4)	48.95±7.74		11.53±2.88		
	>25	42(10.9)	51.62±7.81		12.15±3.17		
Accidents in the last year				0.001		0.018	
	.00	261(68.7)	49.26±6.80		11.02±3.33		
	≥1.00	119(31.3)	46.59±8.15		10.17±3.04		
Driving fines in the last year				0.001		0.004	
	.00	146(38.6)	50.48±6.55		11.25±3.36		
	1.00	54(14.3)	50.48±5.51		11.28±3.39		
	2.00	65(17.2)	47.31±7.92		10.46±2.74		
	3–4	56(14.8)	47.96±6.99		10.80±3.43		
	≥5	57(15.1)	43.16±7.98		9.36±2.95		
Driving hours				0.005		0.464	
	0–0.5	45(12.2)	50.68±5.15		10.58±3.47		
	0.5–1	96(26.0)	49.79±5.47		10.96±3.02		
	1–2	98(26.6)	47.81±7.69		10.96±3.32		
	>2	130(35.2)	46.83±8.91		10.37±3.38		
Driving purpose				0.827			0.553
	More for work	59(15.1)	48.07±7.30		10.36±3.69		
	More personal	105(26.9)	48.79±6.99		10.82±3.39		
	Combination of work and personal	226(57.9)	48.40±7.72		10.87±3.09		

*Missing data was different for the variables measured from zero percent for marital status to 6.4% for certificate type.

According to the multi-factor model, the most important factors related to behavior were age (P<0.001), receiving driving fines (P<0.001), driving hours (P = 0.010), and type of car gearbox (P = 0.007). The most important factors related to behavioral intention were age (P<0.001), and driving fines (P = 0.004) (Table 6).

**Table 6:** Relationship between demographic factors and driving characteristics with behavior and behavioral intention in the multi-factor model.

		Behavior		Behavior intention	
		B±SE	P_value	B±SE	P_value
Age					
	≤25	-6.40±1.26	<0.001	-5.68±1.21	<0.001
	26–35	-4.28±1.03	<0.001	-3.87±1.01	<0.001
	36–45	-3.27±1.07	0.002	-3.40±1.04	0.001
	>45	Ref.		Ref.	Ref.
Gearbox type					
	Automatic	-2.94±1.17	0.007		
	Manual	Ref.			
Driving fine					
	.00	6.82±1.10	<0.001	7.46±1.07	<0.001
	1.00	6.78±1.29	<0.001	6.91±1.28	<0.001
	2.00	3.62±1.24	0.004	3.27±1.24	0.009
	3–4	3.59±1.30	0.006	3.86±1.30	0.003
		Ref.		Ref.	
Driving hours					
	0–0.5h	2.77±1.24	0.027		
	0.5-1h	2.35±0.93	0.012		
	1-2h	0.69±0.91	0.451		
	>2h	Ref.			

### 4.7. Relationship between the TPB construct and moral norm with behavior and behavioral intention in the multi-factor model

In the one-factor model, behavior showed positive significant correlations with attitude (r = 0.442, P = 0.001), perceived behavior control (r = 0.489, P = 0.001), subjective norm (r = 0.344, P = 0.001), behavioral intention (r = 0.446, P = 0.001), and moral norm (r = 0.391, P = 0.001). Additionally, attitude (r = 0.378, P<0.001), perceived behavior control (r = 0.598, P<0.001), subjective norm (r = 0.398, P<0.001), and moral norm (r = 0.269, P<0.001) were positively correlated with behavioral intention (Table 7).

**Table 7:** Correlation between behavior and behavioral intention with the TPB constructs and moral norm.

	Behavior		Behavioral intention	
	Pearson correlation	P_value	Pearson correlation	P_value
Attitude	0.442	0.001	0.378	<0.001
Perceived behavior control	0.489	0.001	0.598	<0.001
Subjective norm	0.344	0.001	0.398	<0.001
Behavioral intention	0.446	0.001	--	--
Moral norm	0.391	0.001	0.269	<0.001

In a multiple-factor model, behavior was found to have a significant relationship with attitude (P<0.001), perceived behavior control (P<0.001), behavioral intention (P = 0.001), and moral norm (P<0.001). These predictors remained significantly correlated with behavior even after controlling for age, receiving driving fines, driving hours, and type of car gearbox. Similarly, perceived behavior control (P<0.001) and subjective norm (P<0.001) were significantly associated with behavioral intention in the multi-factor model. After adjusting for age and driving fines, these factors continued to be related to behavioral intention (Table 8).

**Table 8:** Factors associated with behavior and behavioral intention based on the multi-factor model.

		Behavior				Behavioral intention			
		Model 1*		Model 2**		Model 1*		Model 2**	
		Beta^#^	P_value	Beta	P_value	Beta	P_value	Beta	P_value
Attitude		0.19	<0.001	0.20	<0.001				
Perceived behavior control		0.23	<0.001	0.18	0.001	0.52	<0.001	0.49	<0.001
Subjective norm						0.18	<0.001	0.17	0.002
Behavioral intention		0.18	0.001	0.15	0.003	--			
Moral norm		0.18	<0.001	0.16	<0.001				

* Multiple regression

** Multiple regression after adjusting for significant variables in Table 2

^#^Standardized coefficient

## 5. Discussion

The present study investigated the factors associated with the intention and behavior of MPUWD among the general driving population in Zahedan. The findings indicated that a significant proportion of participants admitted to using their mobile phones while driving. Age, receiving driving fines, driving hours, and type of car gearbox were identified as factors associated with MPUWD. Moreover, the TPB constructs, including attitude, perceived behavior control, behavioral intention, and moral norm, played a significant role in predicting MPUWD. Regarding the intention to use a mobile phone while driving, the study revealed that age, and receiving driving fines were the primary influencing factors. Furthermore, subjective norm and perceived behavior control within the TPB framework also showed associations with the intention of MPUWD.

### 5.1. Frequency of MPUWD

In this study, a significant majority of drivers (86.4%) reported engaging in MPUWD, indicating the widespread prevalence of this risky behavior. This study also investigated the specific activities related to MPUWD. The results revealed that the most common activity reported was answering phone calls, followed by making phone calls, using phone applications, reading text messages, sending text messages, taking pictures, reading social media, answering video calls, posting social media, making video calls, reading emails, and sending emails. In a study conducted in Ukraine, it was reported that 91.6% of Ukrainian drivers engaged in MPUWD, with the most prevalent activities being making and answering phone calls [26]. Similarly, a study conducted in Queensland found that approximately 45% of drivers reported answering phone calls as the most frequent task while driving [19]. Additionally, in a study conducted in Italy, 68.9% of participants admitted to using a cell phone while driving, with answering phone calls being the most common activity, reported by 38% of participants [39].

The prevalence of answering and making phone calls while driving highlights the persistent reliance on voice communication as a primary mode of MPUWD. However, it is concerning that drivers often underestimate the risks associated with engaging in phone conversations while driving [40]. Despite the well-established dangers, drivers continue to participate in these activities. Answering or making phone calls can lead to cognitive and cognitive-visual distractions, diverting drivers’ attention away from the road and significantly increasing the risk of accidents [41].

The study findings highlight the substantial involvement of drivers in the use of phone applications while driving. Interacting with smartphone applications has been shown to introduce substantial distractions, compromising the necessary attention and focus for safe driving in manual, visual, and cognitive aspects [42]. It is important to recognize the potential risks associated with using navigation apps that allow drivers to share police enforcement locations. The audio alerts from these apps may distract drivers, promoting device use while driving, reducing perceived risk, encouraging risky behavior, and undermining the integrity of road rules and enforcement efforts [43]. Additionally, reading text messages emerged as a common behavior among drivers, which poses various distractions, particularly in the visual and manual aspects, diverting their attention from the driving task and increasing the risk of collisions [15].

### 5.2. Factors associated with MPUWD

One of the key findings of this study is the association between various demographic and contextual factors with MPUWD. Age was identified as a significant factor, indicating that younger drivers may be more prone to engaging in this behavior. The study findings indicated a negative association between MPUWD and age, with older drivers demonstrating a lower intention to engage in MPUWD. These findings are consistent with previous research, which has consistently shown that age is a predictor of both intention [44] and frequency of MPUWD [45]. It is not surprising, as young adults are known to be at the highest risk of problematic smartphone use [46]. Moreover, the evidence suggested that as age increases, a higher percentage of respondents perceive the use of mobile phones while driving as "very unsafe." Older drivers are also more likely than younger drivers to support a complete prohibition on cell phone usage while driving [47].

The receipt of police driving fines emerged as another influential factor associated with MPUWD. The study findings indicated a positive association between the number of traffic fines in the last year and both the intention and behavior of MPUWD. This suggests that individuals who have a history of being fined for driving-related offenses, such as speeding or distracted driving, are more likely to intend and engage in MPUWD. One possible explanation for this relationship is the potential lack of significance of the fine amount in Iran, where a fine of 1,000,000 Rials (equivalent to two US dollars in 2022) may have an unintended effect on individual intention and behavior and the relatively low fine amount may not effectively deter individuals from engaging in MPUWD. Research conducted in Queensland, where driving fines are considerably higher than in Iran, revealed that 63% of drivers underestimated the fine for using a phone while driving, while 37% underestimated the demerit points. It was observed that drivers who underestimated phone penalties had a significantly lower perception of punishment severity [48]. Moreover, the findings indicated that individuals who expressed a strong intention to use a mobile phone while driving were less convinced that the risk of fines could effectively deter them from engaging in this risky behavior [49].

This study revealed a significant association between driving hours per day and the behavior of MPUWD, indicating the potential impact of prolonged driving on engagement in risky behaviors such as mobile phone use. Previous research has consistently shown that increasing driving hours per day increases the likelihood of engaging in MPUWD [19]. It is crucial to consider the underlying mechanisms that contribute to this association. Boredom and drowsiness associated with extended periods of driving may prompt drivers to seek secondary tasks, such as mobile phone use, to alleviate these negative states or to engage in social interaction [50].

### 5.3. Relationship between the TPB construct and moral norms with MPUWD behavior and behavioral intention

The present study revealed that attitude played a crucial role in predicting MPUWD, highlighting its significance in comprehending and elucidating this risky behavior. However, it did not exhibit a significant association with the intention of MPUWD. The finding that attitude served as a significant predictor of MPUWD is in line with prior studies [19, 51] and the theoretical framework of the TPB. Attitude represents an individual’s overall assessment or evaluation of the behavior in question, encompassing their beliefs regarding the advantages, risks, and consequences associated with that behavior [24]. Consistent with previous research [39, 52], the notable influence of attitude on MPUWD suggests that individuals’ underlying beliefs and assessments of this behavior have a direct impact on their actual participation in it.

Contrary to expectations, the current study did not find a significant association between attitude and the intention to engage in MPUWD. This finding may appear unexpected since attitude is typically regarded as a strong determinant of behavioral intentions. However, it is important to acknowledge that intention is influenced by various other factors, including subjective norms and perceived behavioral control, which might have overshadowed the impact of attitude within this specific context. In line with a previous study [53], the absence of a significant association between attitude and intention suggests that although individuals may hold certain beliefs and evaluations regarding MPUWD, these attitudes may not necessarily translate into a definite intention to engage in such behavior.

The current study indicates that subjective norm plays a significant role in predicting the intention to use a mobile phone while driving, but it does not have a significant influence on the behavior itself. Consistent with the previous studies [32, 53] the significant association between subjective norm and intention suggests that individuals’ perceptions of social pressure play a role in shaping their intentions to use a mobile phone while driving. If individuals perceive that their significant others approve or expect them to engage in this behavior, they are more likely to form the intention to use a mobile phone while driving. However, it is noteworthy that subjective norms do not demonstrate a significant association with the actual behavior of using a mobile phone while driving. This implies that despite the influence of social pressure on individuals’ intentions, it may not directly translate into their behavior. Other factors, such as perceived behavioral control may come into play when individuals are faced with the actual decision to use a mobile phone while driving. In a previous study examining the relationship between an extended theory of planned behavior and young drivers’ intention and behavior of using social interactive technology while driving in a concealed manner, the results indicated that subjective norm played a significant role in predicting the intention to engage in this behavior among young drivers. However, it did not demonstrate a significant influence on the actual behavior of using social interactive technology while driving [32]. This indicates that although individuals may perceive social pressure to engage in this behavior, they may not always act accordingly.

The present study demonstrated that perceived behavior control was a significant predictor of both the intention and behavior of MPUWD. Consistent with previous findings [30, 53] the results indicate that individuals who perceive themselves to have greater control over their behavior are more likely to form the intention to use a mobile phone while driving. This suggests that individuals’ belief in their ability to control their actions plays a crucial role in shaping their intentions and motivation to engage in the behavior. Moreover, consistent with the previous studies [52] the significant association between perceived behavior control and actual behavior of MPUWD suggests that individuals’ perceived control over their behavior directly influences their engagement in this risky behavior. Individuals who perceive themselves to have a higher level of control are more likely to engage in MPUWD, whereas those who perceive lower control are more likely to refrain from this behavior.

The findings of the present study reveal that moral norm plays a significant role in predicting MPUWD, but interestingly, it does not significantly influence individuals’ intentions to engage in this behavior. The significant association between moral norms and MPUWD suggests that individuals who perceive MPUWD as morally unacceptable are less likely to engage in this behavior. Consistent with the previous studies [23, 37] the finding that moral norm predicts MPUWD highlights the importance of ethical considerations in shaping individuals’ behavior on the road. Emphasizing the ethical aspects of this behavior can create a sense of responsibility and encourage individuals to prioritize the safety of themselves and others. Interestingly, the study did not find a significant association between moral norms and behavioral intention. This result suggests that while moral beliefs strongly influence the actual behavior of MPUWD, they might not have a direct impact on individuals’ intentions to engage in this behavior. This discrepancy may arise from various factors, such as the presence of social desirability bias in self-reported intentions or the influence of other constructs within the TPB framework.

Consistent with previous research [32, 54] the findings of this study highlight the significance of behavioral intention as a predictor of MPUWD. The strong association between behavioral intention and MPUWD suggests that individuals who express a higher intention to engage in this behavior are more likely to use their phones while driving.

Furthermore, the findings of this study demonstrate that attitude, perceived behavior control, behavioral intention, and moral norm play prominent roles in predicting MPUWD after adjusting for other significant variables. Previous research has indicated that these three fundamental variables of the TPB are more influential than demographic variables in predicting the intention of MPUWD [55]. Another study indicated that attitudes, perceived behavioral control, and descriptive norms emerged as significant predictors of self-reported engagement in driver distraction, even after controlling for age group and gender [52]. Consistent with the previous study [55] perceived behavior control emerged as the most important predictor of both intention and behavior of MPUWD, as evidenced by the standardized beta weights.

### 5.4. Limitations

To gain a comprehensive understanding of the implications of this study, it is important to acknowledge its limitations. One limitation is the reliance on self-reported data for assessing MPUWD, which introduces potential biases such as social desirability or recall biases, thereby affecting the reliability of the findings. To mitigate this limitation, future research could incorporate objective measures of mobile phone use, such as smartphone sensor data or in-vehicle monitoring systems. Additionally, utilizing supplementary methodologies like naturalistic driving studies would provide real-time and objective data, enhancing the validity and accuracy of the findings. Another significant limitation of this study is its cross-sectional design, which hinders the ability to establish causality between variables. To address this limitation, future research could adopt longitudinal designs that follow participants over an extended period.

## 6. Conclusion

The findings of this study provide alarming insights into the widespread and diverse nature of MPUWD among the participants. The wide range of behaviors identified emphasizes the complexity of the problem and the need for comprehensive interventions. It is crucial to develop effective interventions that target different forms of MPUWD to address this issue effectively. The study emphasizes the importance of considering demographic factors, driving characteristics, and the TPB constructs in understanding and influencing individuals’ decisions and behaviors related to MPUWD. A thorough understanding of these factors can inform the development of impactful interventions and campaigns aimed at reducing MPUWD and promoting safer driving practices. By addressing these factors comprehensively, we can work towards creating a safer driving environment and minimizing the risks associated with MPUWD.

## Supporting information

S1 Dataset Minimal dataset. (DOCX)
