# Supplementary material for: Different types of mobile phone use while driving and influencing factors on intention and behavior: Insights from an expanded theory of planned behavior
Source: PLoS One. 2024 Mar 6;19(3):e0300158. doi: 10.1371/journal.pone.0300158 (PMC10917291; doi:10.1371/journal.pone.0300158)
Supplement: S1 Dataset — (DOCX) [file pone.0300158.s001.docx]

| Behavior intention | | | | | Behavior | | | | |  | **Table 1** |
| --- | --- | --- | --- | --- | --- | --- | --- | --- | --- | --- | --- |
| significant  P_value | Statisticalmethod used | SE | SD | Mean | significant  P_value | Statistical method used | SE | SD | Mean | #Samples |  |
|  |  |  |  |  |  |  |  |  |  |  | **Age (year)** |
| *P<0.05 | One-factor general linae model (equivalent to one-way ANOVA) | 0.430 | 3.36 | 9.72 | *P<0.05 | One-factor general linae model (equivalent to one-way ANOVA) | 0.896 | 7.00 | 47.20 | 61 | ≤25 |
|  |  | 0.262 | 3.03 | 10.31 |  |  | 0.606 | 7.02 | 47.43 | 134 | 26-35 |
|  |  | 0.309 | 3.30 | 11.04 |  |  | 0.732 | 7.82 | 48.40 | 114 | 36-45 |
|  |  | 0.352 | 3.01 | 12.15 |  |  | 0.779 | 6.66 | 51.90 | 73 | >45 |
|  |  |  |  |  |  |  |  |  |  |  | **Gender** |
| *P<0.05 | One-factor general linae model (equivalent to independent samples T-test) | 0.203 | 3.26 | 10.89 | *P<0.05 | One-factor general linae model (equivalent to independent samples T-test) | 0.483 | 7.77 | 47.91 | 259 | Male |
|  |  | 0.284 | 3.26 | 10.71 |  |  | 0.580 | 6.66 | 49.56 | 132 | Female |
|  |  |  |  |  |  |  |  |  |  |  | **Education** |
| *P<0.05 | One-factor general linae model (equivalent to one-way ANOVA) | 0.529 | 3.04 | 11.90 | *P<0.05 | One-factor general linae model (equivalent to one-way ANOVA) | 1.172 | 6.73 | 51.28 | 33 | under diploma |
|  |  | 0.301 | 2.99 | 10.86 |  |  | 0.617 | 6.14 | 48.79 | 99 | Diploma |
|  |  | 0.262 | 3.45 | 10.54 |  |  | 0.565 | 7.43 | 48.41 | 173 | BSc |
|  |  | 0.343 | 3.18 | 10.76 |  |  | 0.950 | 8.81 | 47.23 | 86 | MSc & PhD |
|  |  |  |  |  |  |  |  |  |  |  | **Marital status** |
| *P<0.05 | One-factor general linae model (equivalent to one-way ANOVA) | 0.293 | 3.13 | 9.89 | *P<0.05 | One-factor general linae model (equivalent to one-way ANOVA) | 0.691 | 7.38 | 47.14 | 114 | Single |
|  |  | 0.197 | 3.25 | 11.18 |  |  | 0.437 | 7.20 | 49.30 | 271 | Married |
|  |  | 1.107 | 2.93 | 9.71 |  |  | 3.508 | 9.28 | 39.14 | 7 | Divorced |
|  |  |  |  |  |  |  |  |  |  |  | **Driving license** |
| *P<0.05 | One-factor general linae model (equivalent to independent samples T-test) | 0.166 | 3.26 | 10.77 | *P<0.05 | One-factor general linae model (equivalent to independent samples T-test) | 0.375 | 7.38 | 48.53 | 387 | Yes |
|  |  | 1.415 | 2.83 | 13.00 |  |  | 6.65 | 13.30 | 43.50 | 4 | No |
|  |  |  |  |  |  |  |  |  |  |  | **Driving certificate type** |
| *P<0.05 | One-factor general linae model (equivalent to one-way ANOVA) | 0.434 | 2.71 | 11.44 | *P<0.05 | One-factor general linae model (equivalent to one-way ANOVA) | 1.206 | 7.53 | 48.21 | 39 | First grade |
|  |  | 0.191 | 3.29 | 10.64 |  |  | 0.433 | 7.47 | 48.53 | 298 | Second grade |
|  |  | 0.608 | 3.33 | 10.77 |  |  | 1.002 | 5.49 | 50.10 | 30 | Third grade |
|  |  |  |  |  |  |  |  |  |  |  | **Gearbox type** |
| *P<0.05 | One-factor general linae model (equivalent to independent samples T-test) | 0.576 | 3.69 | 9.95 | *P<0.05 | One-factor general linae model (equivalent to independent samples T-test) | 1.671 | 10.70 | 45.78 | 41 | Automatic |
|  |  | 0.173 | 3.21 | 10.85 |  |  | 0.368 | 6.83 | 48.83 | 345 | Manual |
|  |  |  |  |  |  |  |  |  |  |  | **Driving experience (year)** |
| *P<0.05 | One-factor general linae model (equivalent to one-way ANOVA) | 0.380 | 3.52 | 10.58 | *P<0.05 | One-factor general linae model (equivalent to one-way ANOVA) | 0.651 | 6.04 | 48.51 | 86 | ≤5 |
|  |  | 0.251 | 3.20 | 10.04 |  |  | 0.602 | 7.68 | 47.20 | 163 | 6-15 |
|  |  | 0.297 | 2.88 | 11.53 |  |  | 0.798 | 7.74 | 48.95 | 94 | 16-25 |
|  |  | 0.489 | 3.17 | 12.15 |  |  | 1.205 | 7.81 | 51.62 | 42 | >25 |
|  |  |  |  |  |  |  |  |  |  |  | **Accidents in the last year** |
| *P<0.05 | One-factor general linae model (equivalent to independent samples T-test) | 0.206 | 3.33 | 11.02 | *P<0.05 | One-factor general linae model (equivalent to independent samples T-test) | 0.421 | 6.80 | 49.26 | 261 | .00 |
|  |  | 0.279 | 3.04 | 10.17 |  |  | 0.747 | 8.15 | 46.59 | 119 | ≥1.00 |
|  |  |  |  |  |  |  |  |  |  |  | **Driving fines in the last year** |
| *P<0.05 | One-factor general linae model (equivalent to one-way ANOVA) | 0.278 | 3.36 | 11.25 | *P<0.05 | One-factor general linae model (equivalent to one-way ANOVA) | 0.542 | 6.55 | 50.48 | 146 | .00 |
|  |  | 0.461 | 3.39 | 11.28 |  |  | 0.750 | 5.51 | 50.48 | 54 | 1.00 |
|  |  | 0.340 | 2.74 | 10.46 |  |  | 0.982 | 7.92 | 47.31 | 65 | 2.00 |
|  |  | 0.458 | 3.43 | 10.80 |  |  | 0.934 | 6.99 | 47.96 | 56 | 3-4 |
|  |  | 0.391 | 2.95 | 9.36 |  |  | 1.057 | 7.98 | 43.16 | 57 | ≥5 |
|  |  |  |  |  |  |  |  |  |  |  | **Driving hours** |
| *P<0.05 | One-factor general linae model (equivalent to one-way ANOVA) | 0.517 | 3.47 | 10.58 | *P<0.05 | One-factor general linae model (equivalent to one-way ANOVA) | 0.768 | 5.15 | 50.68 | 45 | 0-0.5 |
|  |  | 0.308 | 3.02 | 10.96 |  |  | 0.558 | 5.47 | 49.79 | 96 | 0.5-1 |
|  |  | 0.335 | 3.32 | 10.96 |  |  | 0.777 | 7.69 | 47.81 | 98 | 1-2 |
|  |  | 0.296 | 3.38 | 10.37 |  |  | 0.781 | 8.91 | 46.83 | 130 | >2 |
|  |  |  |  |  |  |  |  |  |  |  | **Driving purpose** |
| *P<0.05 | One-factor general linae model (equivalent to one-way ANOVA) | 0.480 | 3.69 | 10.36 | *P<0.05 | One-factor general linae model (equivalent to one-way ANOVA) | 0.950 | 7.30 | 48.07 | 59 | More for work |
|  |  | 0.331 | 3.39 | 10.82 |  |  | 0.682 | 6.99 | 48.79 | 105 | More personal |
|  |  | 0.206 | 3.09 | 10.87 |  |  | 0.514 | 7.72 | 48.40 | 226 | Combination of work and personal |

| Behavior intention | | | | Behavior | | | | **Table 2** |
| --- | --- | --- | --- | --- | --- | --- | --- | --- |
| significant  P_value | Statistical methods used | SE | B | significant  P_value | Statistical methods used | SE | B |  |
|  |  |  |  |  |  |  |  | **Age (year)** |
| *P<0.05 | Multi-factor general linear model (equivalent to factorial design ANOVA model) | 1.21 | -5.68 | *P<0.05 | Multi-factor general linear model (equivalent to factorial design ANOVA model) | 1.26 | -6.40 | ≤25 |
|  |  | 1.01 | -3.87 |  |  | 1.03 | -4.28 | 26-35 |
|  |  | 1.04 | -3.40 |  |  | 1.07 | -3.27 | 36-45 |
|  |  |  | 0 (Ref.) |  |  |  | 0 (Ref.) | >45 |
|  |  |  |  |  |  |  |  | **Gearbox type** |
|  |  |  |  | *P<0.05 | Multi-factor general linear model (equivalent to factorial design ANOVA model) | 1.17 | -2.94 | Automatic |
|  |  |  |  |  |  |  | 0 (Ref.) | Manual |
|  |  |  |  |  |  |  |  | **Driving fine** |
| *P<0.05 | Multi-factor general linear model (equivalent to factorial design ANOVA model) | 1.07 | 7.46 | *P<0.05 | Multi-factor general linear model (equivalent to factorial design ANOVA model) | 1.10 | 6.82 | .00 |
|  |  | 1.28 | 6.91 |  |  | 1.29 | 6.78 | 1.00 |
|  |  | 1.24 | 3.27 |  |  | 1.24 | 3.62 | 2.00 |
|  |  | 1.30 | 3.86 |  |  | 1.30 | 3.59 | 3-4 |
|  |  |  | 0 (Ref.) |  |  |  | 0 (Ref.) |  |
|  |  |  |  |  |  |  |  | **Driving hours** |
|  |  |  |  | *P<0.05 | Multi-factor general linear model (equivalent to factorial design ANOVA model) | 1.24 | 2.77 | 0-0.5h |
|  |  |  |  |  |  | 0.93 | 2.35 | 0.5-1h |
|  |  |  |  |  |  | 0.91 | 0.69 | 1-2h |
|  |  |  |  |  |  |  | 0 (Ref.) | >2h |

| **Table 3** | #samples | Behavior | | | Behavior intention | | |
| --- | --- | --- | --- | --- | --- | --- | --- |
|  |  | Pearson correlation | Statistical method used | significant  P_value | Pearson correlation | Statistical method used | significant  P_value |
| Attitude | 392 | 0.442 | Pearson's correlation test | *P<0.05 | 0.378 | Pearson's correlation test | *P<0.05 |
| Perceived behavior control | 392 | 0.489 | Pearson's correlation test | *P<0.05 | 0.598 | Pearson's correlation test | *P<0.05 |
| Subjective norm | 392 | 0.344 | Pearson's correlation test | *P<0.05 | 0.398 | Pearson's correlation test | *P<0.05 |
| Behavior intention | 392 | 0.446 | Pearson's correlation test | *P<0.05 | -- | Pearson's correlation test | -- |
| Moral norm | 392 | 0.391 | Pearson's correlation test | *P<0.05 | 0.269 | Pearson's correlation test | *P<0.05 |

| **Table 4** | Behavior | | | | Behavior intention | | | |
| --- | --- | --- | --- | --- | --- | --- | --- | --- |
|  | Multiple regression | | Multiple regression after adjusting for age, gearbox type, driving fine, and driving hours | | Multiple regression | | Multiple regression after adjusting for age, and driving fine | |
|  | Standarized Beta | significant  P_value | Standarized Beta | significant  P_value | Standarized Beta | significant  P_value | Standarized Beta | significant  P_value |
| Attitude | 0.19 | *P<0.05 | 0.20 | *P<0.05 |  |  |  |  |
| Perceived behavior control | 0.23 | *P<0.05 | 0.18 | *P<0.05 | 0.52 | *P<0.05 | 0.49 | *P<0.05 |
| Subjective norm |  |  |  |  | 0.18 | *P<0.05 | 0.17 | *P<0.05 |
| Behavior intention | 0.18 | *P<0.05 | 0.15 | *P<0.05 | --- |  |  |  |
| Moral norm | 0.18 | *P<0.05 | 0.16 | *P<0.05 |  |  |  |  |

| **Table 6** | Age (year) | | | | | | | |  |  |  |  |
| --- | --- | --- | --- | --- | --- | --- | --- | --- | --- | --- | --- | --- |
|  | ≤25  (n=61) | | 26-35  (n=134) | | 36-45  (n=114) | | >45  (n=73) | | Statistical method used | P_value | Statistical method used | P_value |
|  | Mean | SD | Mean | SD | Mean | SD | Mean | SD |  |  |  |  |
| Using a mobile phone while driving | 2.72 | 0.71 | 2.63 | 0.78 | 2.69 | 0.79 | 3.11 | 0.68 | One-way ANOVA | *P<0.05 | Trend test for mean | *P<0.05 |
| Answering phone calls while driving | 2.33 | 1.01 | 2.37 | 0.98 | 2.42 | 0.88 | 2.93 | 0.92 | One-way ANOVA | *P<0.05 | Trend test for mean | *P<0.05 |
| Making phone calls while driving | 2.58 | 0.97 | 2.72 | 0.90 | 2.70 | 0.93 | 3.31 | 0.78 | One-way ANOVA | *P<0.05 | Trend test for mean | *P<0.05 |
| Reading messages while driving | 3.10 | 0.81 | 3.16 | 0.79 | 3.26 | 0.94 | 3.53 | 0.82 | One-way ANOVA | *P<0.05 | Trend test for mean | *P<0.05 |
| Sending messages while driving | 3.33 | 0.80 | 3.33 | 0.74 | 3.38 | 0.94 | 3.74 | 0.67 | One-way ANOVA | *P<0.05 | Trend test for mean | *P<0.05 |
| Reading emails while driving | 3.82 | 0.54 | 3.87 | 0.52 | 3.77 | 0.66 | 3.89 | 0.46 | One-way ANOVA | *P<0.05 | Trend test for mean | *P<0.05 |
| Sending emails while driving | 3.88 | 0.46 | 3.86 | 0.53 | 3.80 | 0.60 | 3.90 | 0.42 | One-way ANOVA | *P<0.05 | Trend test for mean | *P<0.05 |
| Reading /viewing social media (e.g., Instagram, WhatsUp, Telegram) posts while driving | 3.57 | 0.76 | 3.46 | 0.72 | 3.64 | 0.72 | 3.82 | 0.56 | One-way ANOVA | *P<0.05 | Trend test for mean | *P<0.05 |
| Posting on social media (e.g., Instagram, WhatsUp, Telegram) while driving | 3.62 | 0.82 | 3.59 | 0.69 | 3.65 | 0.69 | 3.85 | 0.57 | One-way ANOVA | *P<0.05 | Trend test for mean | *P<0.05 |
| Answering video calls while driving | 3.59 | 0.74 | 3.50 | 0.85 | 3.57 | 0.82 | 3.81 | 0.57 | One-way ANOVA | *P<0.05 | Trend test for mean | *P<0.05 |
| Making video calls while driving | 3.61 | 0.77 | 3.52 | 0.80 | 3.68 | 0.74 | 3.88 | 0.56 | One-way ANOVA | *P<0.05 | Trend test for mean | *P<0.05 |
| Using phone applications (e.g., map) while driving | 2.78 | 0.86 | 2.84 | 0.87 | 2.96 | 0.89 | 3.44 | 0.76 | One-way ANOVA | *P<0.05 | Trend test for mean | *P<0.05 |
| Taking pictures of yourself, others, or scenery while driving | 3.37 | 0.71 | 3.43 | 0.80 | 3.59 | 0.69 | 3.74 | 0.65 | One-way ANOVA | *P<0.05 | Trend test for mean | *P<0.05 |
| Turning off the phone while driving | 3.87 | 0.34 | 3.65 | 0.66 | 3.65 | 0.64 | 3.59 | 0.70 | One-way ANOVA | *P<0.05 | Trend test for mean | *P<0.05 |
| Stop driving to answer received phone calls or messages | 2.85 | 0.90 | 2.83 | 0.88 | 2.76 | 0.98 | 2.64 | 1.04 | One-way ANOVA | *P<0.05 | Trend test for mean | *P<0.05 |
| Using headphones while driving | 3.28 | 0.90 | 3.29 | 0.87 | 3.35 | 0.79 | 3.78 | 0.48 | One-way ANOVA | *P<0.05 | Trend test for mean | *P<0.05 |

There was 10 missing values for age

| **Table 8** | Age (year) | | | | | | | |  |  |  |  |
| --- | --- | --- | --- | --- | --- | --- | --- | --- | --- | --- | --- | --- |
|  | ≤25  (n=61) | | 26-35  (n=134) | | 36-45  (n=114) | | >45  (n=73) | | Statistical methods used | P_value | Statistical methods used | P_value |
|  | Mean | SD | Mean | SD | Mean | SD | Mean | SD |  |  |  |  |
| I intend to use my mobile phone while driving next week | 3.25 | 0.98 | 3.41 | 1.06 | 3.49 | 1.18 | 3.56 | 1.17 | One-way ANOVA | *P<0.05 | Trend test for mean | *P<0.05 |
| It is more likely to use my mobile phone while driving next week | 3.90 | 1.13 | 3.62 | 1.13 | 3.61 | 1.18 | 3.85 | 1.22 | One-way ANOVA | *P<0.05 | Trend test for mean | *P<0.05 |
| I expect to use my mobile phone while driving next week | 3.52 | 1.11 | 3.46 | 1.08 | 3.72 | 1.18 | 3.74 | 1.16 | One-way ANOVA | *P<0.05 | Trend test for mean | *P<0.05 |

There was 10 missing values for age
